# Antitumor effects and potential mechanisms of aconitine based on preclinical studies: an updated systematic review and meta-analysis

**DOI:** 10.3389/fphar.2023.1172939

**Published:** 2023-04-27

**Authors:** Gelin Xiang, Nan Xing, Shaohui Wang, Yi Zhang

**Affiliations:** ^1^ State Key Laboratory of Southwestern Chinese Medicine Resources, School of Ethnic Medicine, Chengdu University of Traditional Chinese Medicine, Chengdu, China; ^2^ State Key Laboratory of Southwestern Chinese Medicine Resources, Research Center for Academic Inheritance and Innovation of Ethnomedicine, Chengdu University of Traditional Chinese Medicine, Chengdu, China; ^3^ State Key Laboratory of Southwestern Chinese Medicine Resources, School of Pharmacy, Chengdu University of Traditional Chinese Medicine, Chengdu, China

**Keywords:** aconitine, malignancy, preclinical studies, meta-analysis, bax, NF-κB, bcl-2

## Abstract

**Background:** Herbs originating from the *Aconitum* L. (Ranunculaceae), such as *Aconitum carmichaelii* Debeaux. (Wutou), *Aconitum pendulum* Busch. (Tiebangchui), and *Aconitum kusnezoffii* Reichb. (Caowu), etc. are highly valued for their medicinal properties. The roots and tubers of these herbs are commonly used to treat an array of ailments, including joint pain and tumors. The alkaloids present in them are the primary active components, with aconitine being the most notable. Aconitine has gained attention for its exceptional anti-inflammatory and analgesic properties, as well as its potential as an anti-tumor and cardiotonic agent. However, the exact process through which aconitine hinders the growth of cancerous cells and triggers their programmed cell death remains unclear. Therefore, we have undertaken a comprehensive systematic review and meta-analysis of the current research on the potential antitumor properties of aconitine.

**Methods:** We conducted a thorough search of relevant preclinical studies in databases including PubMed, Web of Science, VIP, WanFang Data, CNKI, Embase, Cochrane Library, and National Center for Biotechnology Information (NCBI). The search was conducted up until 15 September 2022, and the data were statistically analyzed using RevMan 5.4 software. The number of tumor cell value-added, tumor cell apoptosis rate, thymus index (TI), and Bcl-2 gene expression level were the main indicators to be analyzed.

**Results:** After applying the final inclusion criteria, a total of thirty-seven studies, comprising both *in vivo* and *in vitro* research were analyzed. The results showed that treatment with aconitine led to a significant reduction in tumor cell proliferation, a noteworthy increase in the rate of apoptosis among tumor cells, a decrease in the thymus index, and a reduction in the expression level of Bcl-2. These results suggested that aconitine could inhibit the proliferation, invasion, and migration abilities of tumor cells by regulating Bcl-2 etc., thereby enhancing the anti-tumor effects.

**Conclusion:** In summary, our present study demonstrated that aconitine effectively reduced tumor size and volume, indicating a strong anti-tumor effect. Additionally, aconitine could increase the expression levels of caspase-3, Bax and other targets. Mechanistically, it may regulate the expression levels of Bax and Bcl-2 through the NF-κB signaling pathway, ultimately inhibiting tumor cell proliferation through autophagy.

## 1 Introduction

**FIGURE 1:**
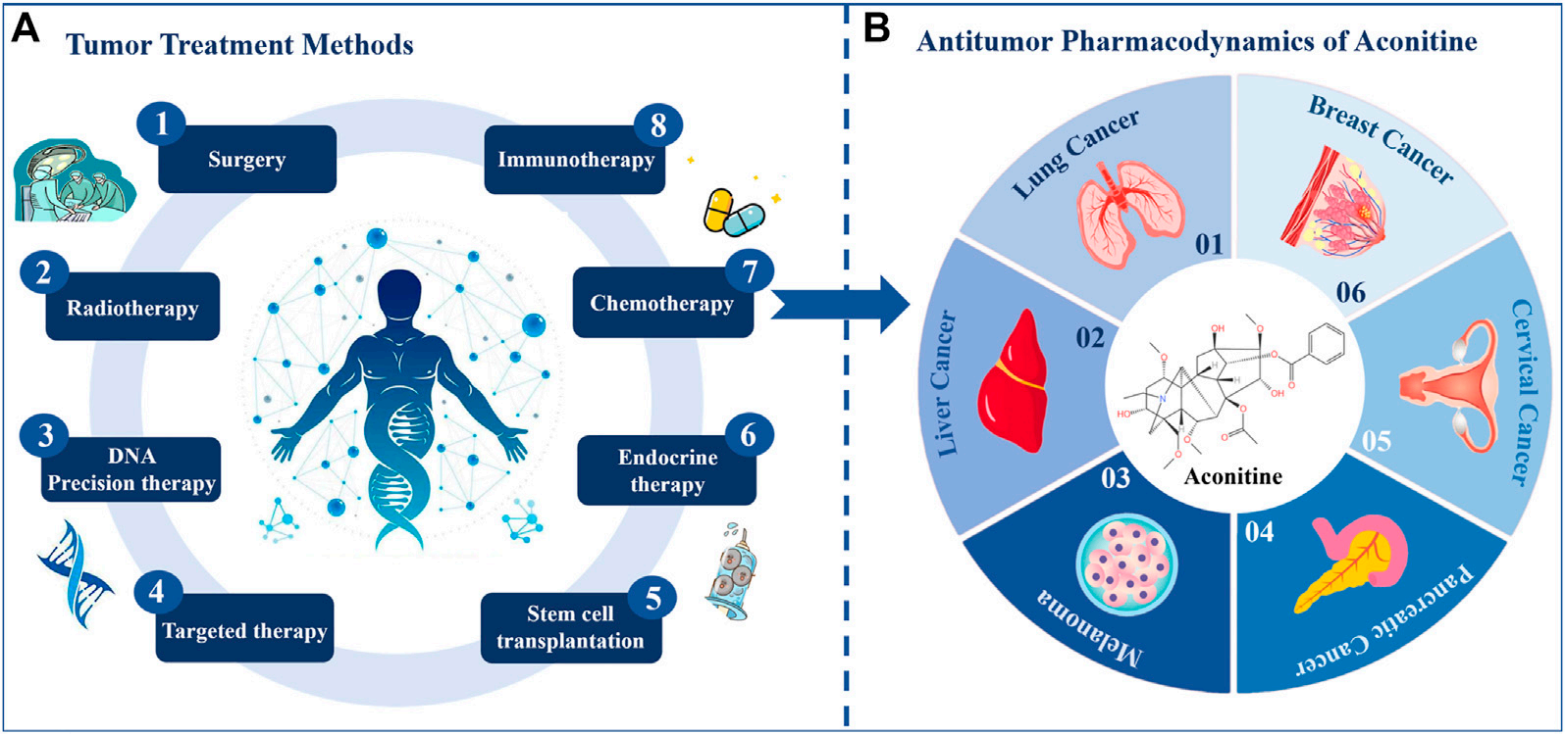
The main method of tumor treatment. **(A)** Tumor treatment methods. **(B)** Antitumor pharmacodynamics of aconitine.

A malignant tumor refers to a vast array of diseases that are characterized by the loss of normal cellular regulation, uncontrolled growth, abnormal differentiation, local tissue infiltration, and distant transplantation (Reardon, 2010). Malignant tumors can develop in any tissue of any organ and can occur at any age (Klein and Vande, 2008). The International Agency for Research on Cancer (IARC) of the World Health Organization has released the latest global cancer burden data, revealing that there will be 19.29 million new cancer cases worldwide in 2020. Shockingly, 4.57 million of these cases will be in China, accounting for 23.7% of the new global cancer cases. To make matters worse, both the number of cancer deaths and the number of new cancers globally rank first in the world (Zhang, 2021). In the field of oncology, there are several options available to treat malignant tumors. These include surgery, radiotherapy (Baskar et al., 2012), chemotherapy, immunotherapy, targeted therapy (Guan et al., 2018), endocrine therapy (Reinbolt et al., 2015), stem cell transplantation (Hawsawi et al., 2018), and DNA precision therapy (Raimundo et al., 2021). However, two of the most commonly used treatments, chemotherapy and radiotherapy, often come with a range of side effects (Tommelein et al., 2018), such as damage to local radiation areas, hair loss, nausea and vomiting, fever, and impaired hematopoietic function. Additionally, oral drugs used in treatment can also lead to reduced patient wellbeing due to associated side effects (Lin et al., 2017; Ding and Lu, 2019). With targeted therapy, the required drugs are taken for a long time and the specific drugs are expensive. Therefore, it is crucial to find alternative drugs for the treatment of malignant tumors. The current treatment modalities for malignancies are shown in Figure 1A. Chinese herbal medicine has become a Frontier area of oncology drug research because of its lesser side effects and unique pharmacological activities. More and more scholars have started to search for natural products with antitumor efficacy from herbal medicines.

**FIGURE 2:**
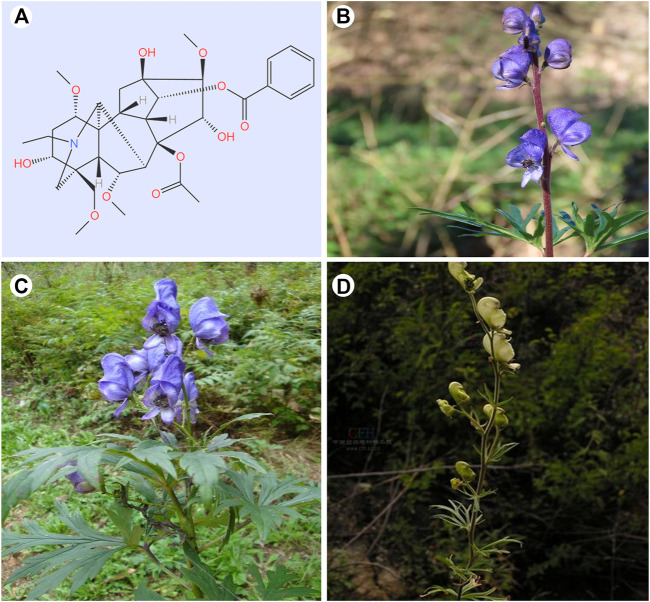
Structural formula of aconitine and botanical diagram of some major *Aconitum* source species. **(A)** The structural formula of Aconitine. **(B)** *Aconitum carmichaelii* Debeaux. (Wutou). **(C)** *Aconitum brachypodum* Diels. (Xueshangyizhihao). **(D)** *Aconitum coreanum* (H. Lév.) Raipaics. (Huanghuawutou). The representative photos of the three *Aconitum* species are from the website: https://www.gbif.org/; http://powo.science.kew.org.

Medicinal materials derived from *Aconitum* L., including *Aconitum carmichaelii* Debeaux. (Wutou), *Aconitum brachypodum* Diels. (Xueshangyizhihao), *Aconitum coreanum* (H. Lév.) Raipaics. (Huanghuawutou), *Aconitum kusnezoffii* Reichb. (Caowu), and *Aconitum pendulum* Busch. (Tiebangchui), etc. are well-known Chinese herbal medicines. It is reported to be widely used in many traditional medical systems, including Chinese, Tibetan, Mongolian, and Indian medicine. Modern pharmacological research has confirmed their analgesic and anti-tumor effects, which has sparked interest among scholars in the field of oncology (Ma et al., 2015; Li et al., 2022) (Figure 2). Aconitine (C_34_H_47_NO_11_) is a C_19_-diterpenoid alkaloid that possesses good anti-tumor effects and is the main medicinal ingredient in *Aconitum* medicinal materials (Yang et al., 2016) (Figure 1B). Clinically, aconitine is mostly used to prevent and treat cancer, including pancreatic cancer (Ji et al., 2016), ovarian cancer (Li et al., 2018), breast cancer (Guo et al., 2011), lung cancer (Zhang et al., 2020), liver cancer (Qi et al., 2018a; Yao et al., 2021) and melanoma (Du J et al., 2013), etc. Its action mechanism is primarily to induce apoptosis, inhibit cancer cell proliferation and migration (Garmanchouk et al., 2005; Wang et al., 2020; Wang et al., 2022), etc. In addition, it is also used for local anesthesia (Chan et al., 2021), analgesia (Wang et al., 2008), anti-inflammation (Zheng et al., 2017), and sweating (Wu et al., 2021; Zhang et al., 2021). Aconitine has shown excellent efficacy in anti-inflammation, for instance, in the treatment of rheumatoid arthritis, by regulating IL-6 and TNF-α cytokine levels and inhibiting the activation of NF-κB signaling pathway (Chen et al., 2021). Currently, several animal and cellular experiments have confirmed the inhibitory effect of aconitine alkaloids on malignant tumors (Yao et al., 2021; Li et al., 2022). As shown above, aconitine shows powerful antitumor potential in a variety of tumors and has good prospects for development and application.

Although aconitine has anti-inflammatory and analgesic properties, improper use can result in severe cardiac arrhythmias, shock, and coma (Gao et al., 2020). This is because aconitine affects voltage-sensitive sodium channels in excitable tissues such as cardiac muscle, nerve, and muscle, leading to cardiotoxicity and neurotoxicity (Yang et al., 2021; Gao et al., 2022; Jiang et al., 2022). Furthermore, studies in recent years have also shown that aconitine can cause embryotoxicity (Li et al., 2020), nephrotoxicity (Jin et al., 2020), hepatotoxicity (Ji et al., 2019), and reproductive toxicity (Wang et al., 2019). Numerous preclinical studies have demonstrated that aconitine has a notable inhibitory effect on malignant tumors. However, these results originate from various laboratories and users, which may result in some inaccuracies. As a result, it is essential to integrate and analyze the study outcomes using appropriate methods.

Systematic review is a highly effective method of producing dependable information that can serve as the most authoritative form of medical evidence. According to the Oxford Center for Evidence-Based Medicine grades of evidence, only data from systematic reviews can be recognized as 1A evidence (Glasziou et al., 2004). As a generally accepted and effective method, the search for aconitine’s extensive preclinical evidence of inhibiting malignant tumors and promoting apoptosis of malignant tumor cells *in vitro* and *in vivo* can help expand the clinical application of aconitine and the subsequent development of related anticancer drugs. In this study, a systematic evaluation and meta-analysis of aconitine’s antitumor properties were conducted for the first time. This involved searching both domestic and international literature databases to identify relevant preclinical literature and to determine the anti-tumor mechanism of aconitine. The aim was to provide a systematic evaluation that would serve as an evidence-based foundation for subsequent anti-tumor research on aconitine.

## 2 Materials and methods

### 2.1 Protocol

This meta-analysis strictly adhered to the protocol registered in PROSPERO (CRD42022370809) and followed the PRISMA guidelines.

### 2.2 Retrieval strategy

In this subject paper, we utilized various computerized search databases such as WanFang Data, CNKI, VIP, PubMed, Embase, Web of Science, and Cochrane Library. The search period was limited to the date of creation up to 15 September 2022. Additionally, to ensure that we did not miss any relevant articles, we conducted a manual search of the retrieved articles.

#### 2.2.1 Chinese database retrieval strategy

The Chinese literature search was conducted through the following search strategy: ① “aconitine” or “aconitine alkaloid”; ② “anti-cancer” or “anti-tumor” or “anti-proliferation” or “anti-proliferative” or “anti-proliferative activity” or “inhibition of cell proliferation”; ③ “cancer cell” or “tumor cell”; ④ including ①②③ and other subject words or free words.

The search strategy for Chinese literature involved the following steps: ①, searching for keywords “aconitine” or “aconitine alkaloid”; ②, looking for terms such as “anti-cancer” or “anti-tumor” or “anti-proliferation” or “anti-proliferative” or “anti-proliferative activity” or “inhibition of cell proliferation”; ③, searching for phrases like “cancer cell” or “tumor cell”; ④, including ①②③ along with other relevant subject words or free words.

#### 2.2.2 English database retrieval strategy

To search for English literature on the topic, the following strategy was employed: ①, Keywords such as “Aconitine” or “Aconitum alkaloid” or “Aconitine alkaloids” were used; ②, Terms such as “Anticancer” or “Antitumor” or “Antiproliferative” “Anti-proliferation” or “Antiproliferative activity” were included; ③, Phrases like “Cancer cell” or “Tumor cell” were also added; ④, All the above terms were combined using “OR” and “AND” to form a comprehensive search group. In addition, other relevant subject words and free words were included.

These were the techniques we used to conduct our literature searches: [“aconitine” (MeSH Terms) OR “aconitine” (All Fields)] OR [“aconitum” (MeSH Terms) OR “aconitum” (All Fields)] AND [“alkaloids” (MeSH Terms) OR “alkaloids” (All Fields) OR “alkaloid” (All Fields)] OR [“aconitine” (MeSH Terms) OR “aconitine” (All Fields)] AND [“alkaloids” (MeSH Terms) OR “alkaloids” (All Fields)] AND [Anticancer (All Fields) OR Antitumor (All Fields) OR Antiproliferative (All Fields) OR Anti-proliferation (All Fields)] OR [Antiproliferative (All Fields)] AND [“motor activity” (MeSH Terms)] OR [“motor” (All Fields) AND “activity” (All Fields)] OR [“motor activity” (All Fields) OR “activity” (All Fields)] AND [“cancer” (All Fields) AND “cell” (All Fields)] OR [“cancer cell” (All Fields)] OR [“tumour” (All Fields) OR “neoplasms” (MeSH Terms) OR “neoplasms” (All Fields) OR “tumor” (All Fields)] AND [“cells” (MeSH Terms) OR “cells” (All Fields) OR “cell” (All Fields)].

### 2.3 Literature inclusion and exclusion criteria

#### 2.3.1 Inclusion criteria

1) Study: Aconitine inhibits malignant tumor cell proliferation and promotes tumor cell apoptosis related article. 2) The experimental group received aconitine monotherapy without regard to treatment method, dose and frequency. 3) The control group was given only saline, pure water or no adjuvant intervention. 4) Outcome indicators: the main outcome indicators included tumor weight, tumor cell inhibition rate (IR), tumor cell apoptosis rate, thymus index (TI), and degree of apoptotic invasion, which encompassed at least one of the above indicators.

#### 2.3.2 Exclusion criteria

1) The target disease was not malignancy, cancer, etc.; 2) There was no control group; 3) The trial group received a combination of aconitine; 4) Duplicate published literature; 5) Studies were clinical studies, case reports, clinical trials, reviews, conference papers, abstracts, reviews, and patent results; 6) Unpublished dissertations; and 7) Literature for which data could not be extracted.

#### 2.3.3 Literature screening and data extraction

Two investigators conducted literature screening and data extraction independently, utilizing Endnote and adhering to the aforementioned inclusion and exclusion criteria. The extracted data will be organized using Excel software and cross-checked for accuracy. In the event of any discrepancies, a joint decision will be made after consulting with a third researcher. Further details regarding the data extraction process can be found below: 1) The first author and year of publication of the literature; 2) Individual data of the experimental study, such as animal species, sex, body weight, and individual comparison samples; 3) The type of anesthesia; 4) The intervention characteristics of the treatment and control groups, including drug dosage form, dose, treatment method, and frequency of administration; and 5) The mean, standard deviation, and between-group differences of measurements and corresponding data.

#### 2.3.4 Quality evaluation of included documents

Two authors conducted independent assessments of the methodological quality of the studies included in this paper. The assessments were based on the CAMARADES list, which provides a collaborative approach to meta-analysis and review of animal data from experimental studies. The list comprises 10 items (Sena et al., 2007): ①Publication of the paper after peer review; ② Description of temperature control; ③ Randomization to treatment and control groups; ④ Blinding of the model; ⑤ Blinded assessment of the results; ⑥ No intrinsic neuroprotective effect of the use of anesthetics; ⑦ Appropriate animal models; ⑧ Sample size calculation; ⑨ Compliance with animal protection regulations, and ⑩ Declaration of any potential conflicts of interest. Each project was rated for overall quality using a single-point system. In the event of any discrepancies in the quality assessment, a third investigator was consulted to resolve them.

#### 2.3.5 Statistical methods

Meta-analysis was performed by using Review Manager 5.4 software. Odds ratios (OR) were used to express count data as statistical effect sizes, while mean difference (MD) was used to express continuous variables. To ensure accuracy, all effect sizes were accompanied by a 95% confidence interval (CI). Heterogeneity was evaluated using Higgins *I* ^2^, and when the results of the heterogeneity test were not statistically significant (*p* > 0.05, *I* ^2^ ≤ 50%), a fixed-effects model was employed for meta-analysis, However, if *I* ^2^ > 50%, a heterogeneity test was required to identify the cause of heterogeneity. If heterogeneity remained above 50% after sensitivity analysis or subgroup analysis, only a description of the results was required. The results were considered statistically significant with a *p*-value of *p <* 0.05. Meta-analysis test level was *a* = 0.05, and publication bias was expressed using funnel plots.

## 3 Results

### 3.1 Study inclusion

**FIGURE 3:**
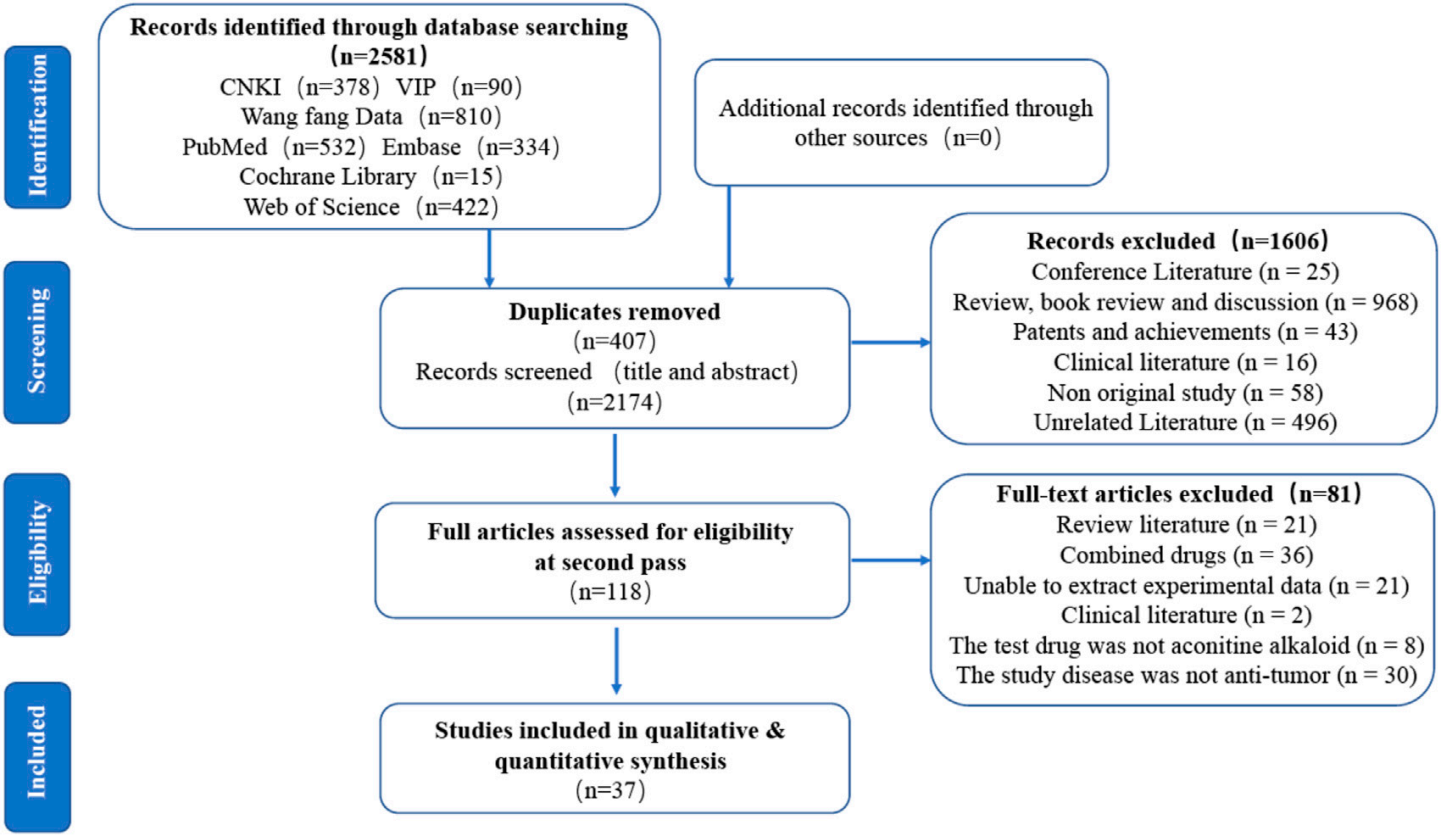
Flow chart of the database search and study identification.

Based on the search method described above, a total of 2581 documents were initially searched. After removing 407 duplicate documents, 2174 articles remained. Of these, 1606 articles were excluded as they were reviews, experiences, conferences, patents, or results, leaving 568 articles after the initial screening. After reviewing the titles and abstracts of these articles, 450 papers were found to have inconsistent contents and were excluded, resulting in a final selection of 118 papers. After reading the full text, we excluded several papers. Specifically, we excluded 21 papers that were reviewed, 36 papers that involved combined drug interventions in both the treatment and control groups, 21 papers from which we were unable to extract experimental data, 2 clinical papers, 8 papers that were tested with drugs other than aconitine alkaloids, and 30 papers that studied diseases other than antitumor. Ultimately, we included 37 literatures for analysis. The screening process is shown in Figure 3. Included literature is shown in Table 1.

**TABLE 1:** Inclusion of literature information.

No.	Author	Time	Source/Nature	Experiment type (animal/cell)	Tumor/Cell type	Database sources	Ref.
1	Tang et al.	1986	Unknown	Cell Experiment	Stomach Cancer	CNKI	Tang and Sun (1986)
2	Yang et al.	2005	Unknown	Animal and Cell Experiment	Lung Cancer	CNKI, WanFang, VIP	Yang et al. (2005)
3	Zeng et al.	2007	Unknown	Animal and Cell Experiment	LoVo, MGC-803, S_180_ tumor cells	CNKI, WanFang, VIP	Zeng et al. (2007)
4	Ren et al.	2008	Fuzi	Animal Experiment	Liver Cancer	CNKI, VIP	Ren and Zeng (2008)
5	Wu et al.	2008	*Folium Aconiti Kusnezoffii* Reichb	Animal Experiment	Monocyte-macrophage	CNKI, WanFang, VIP	Wuliji et al. (2008)
6	Xu et al.	2008	*Aconitum vaginatum* Pritz	Cell Experiment	S_180_ tumor cells	CNKI, WanFang, VIP	Xu et al. (2008)
7	Zhu	2008	*Aconitum vaginatum* Pritz	Cell Experiment	Gastric cancer, Liver cancer, Lung cancer	CNKI, WanFang	Zhu (2008)
8	Rao et al.	2010	Unknown	Cell Experiment	Retinal nerve cells	CNKI, WanFang	Rao and Peng (2010)
9	Jia et al.	2011	Unknown	Cell Experiment	Stomach Cancer	CNKI, WanFang, VIP	Jia and Zhang (2011)
10	Zhang et al.	2011	Fuzi	Cell Experiment	Stomach Cancer	CNKI, WanFang, VIP	Zhang and Wu (2011)
11	Ding et al.	2013	Fuzi	Cell Experiment	Stomach Cancer	CNKI, WanFang, VIP	Ding et al. (2013)
12	Liu	2013	*Aconitum leucostomum* Worosch	Cell Experiment	Leukemia K562 cells	CNKI, WanFang	Liu (2013)
13	Ding	2014	Fuzi	Cell Experiment	Stomach Cancer	CNKI	Ding (2014)
14	Hao	2014	*Aconitum flavum* Hand	Cell Experiment	Gastric cancer, Liver Cancer, Lung cancer	CNKI, WanFang	Hao (2014)
15	Zhao et al.	2014	Unknown	Animal and Cell Experiment	Lung Cancer	CNKI, VIP	Zhao et al. (2014)
16	Fan et al.	2015	*Aconitum szechenyianum* Gay	Cell Experiment	Kidney cells	PubMed, Embase, Web of Science	Fan et al. (2016)
17	Guan et al.	2015	Unknown	Cell Experiment	Leukemia K562 cells	CNKI, WanFang, VIP	Guan et al. (2015)
18	Ji et al.	2016	Unknown	Animal and Cell Experiment	Pancreatic Cancer	PubMed, Embase	Ji et al. (2016)
19	Guan et al.	2017	Unknown	Cell Experiment	Leukemia K562 cells	CNKI, WanFang, VIP	Guan et al. (2017)
20	Ma	2017	Unknown	Cell Experiment	Lung Cancer	CNKI, WanFang	Ma (2017)
21	Zhang et al.	2017	Unknown	Animal Experiment	Lung Cancer	PubMed, Embase, Web of Science	Zhang et al. (2017)
22	Ma et al.	2018	Unknown	Cell Experiment	Myeloma cells	CNKI, WanFang, VIP	Ma and Yu (2018)
23	Qi et al.	2018	Unknown	Animal and Cell Experiment	Liver Cancer	PubMed, Web of Science	Qi et al. (2018b)
24	Wu et al.	2018	Unknown	Animal and Cell Experiment	Breast Cancer	PubMed, Embase, Web of Science	Wu et al. (2018)
25	Xiong et al.	2018	Unknown	Cell Experiment	Liver Cancer	CNKI, WanFang, VIP	Xiong et al. (2018)
26	Zhang et al.	2018	Fuzi	Cell Experiment	H9c2 tumor cells	CNKI, WanFang, VIP	Zhang et al. (2018)
27	Cheng	2019	Unknown	Animal and Cell Experiment	Stomach Cancer	CNKI	Cheng (2019)
28	Shao et al.	2019	Unknown	Cell Experiment	Lung Cancer	CNKI, WanFang, VIP	Shao et al. (2019)
29	Zhou et al.	2019	Unknown	Cell Experiment	Esophageal Cancer	CNKI, WanFang, VIP	Zhou et al. (2019)
30	Cheng et al.	2020	Fuzi	Cell Experiment	Regulation of T cells	CNKI, WanFang, VIP	Cheng et al. (2020)
31	Ru et al.	2020	Unknown	Cell Experiment	Stomach Cancer	CNKI, WanFang, VIP	Ru et al. (2020)
32	Wang	2020	Unknown	Animal Experiment	Liver Cancer	CNKI, WanFang	Wang (2020)
33	Wang et al.	2020	Unknown	Cell Experiment	Ovarian Cancer	PubMed, Web of Science	Wang et al. (2020)
34	Zhang et al.	2020	Unknown	Cell Experiment	Lung Cancer	CNKI, VIP	Zhang et al. (2020)
35	Zou et al.	2021	Unknown	Cell Experiment	Osteosarcoma cells	CNKI, WanFang, VIP	Zou et al. (2021)
36	Cai	2022	Unknown	Cell Experiment	Prostate Cancer	CNKI, WanFang, VIP	Cai (2022)
37	Luan et al.	2022	Unknown	Animal and Cell Experiment	Breast Cancer	PubMed, Embase	Luan et al. (2022)

Note, Fuzi is the root of *Aconitum carmichaelii* Debeaux. (Wutou).

### 3.2 Basic characteristics of included literature

The subject paper included thirty-seven preclinical studies, which could be classified into five animal experiments, twenty-four cellular experiments, and eight experiments that involved both animal and cellular testing. The animal experiments involved a combination of male and female animals in four studies, while eight studies used only male animals and one study used only female animals. Additionally, thirteen animal studies provided information on the body weight of the animals. Eight studies did not report the method of animal execution, while four studies utilized decerebrate execution and one study used 0.1 mL of 1% pentobarbital solution for anesthetic execution. The dosing time ranged from a minimum of 5 days to a maximum of 26 weeks. In terms of cellular experiments, fifteen studies mentioned the cell inhibition rate or cell proliferation inhibition rate. Sixteen studies reported on apoptosis rate, while six studies focused on detecting rate OD or absorbance A value. Additionally, five studies provided insight into different cell cycle numbers, while six studies explored cell invasion ability, cell invasion inhibition rate, or cell invasion number. Furthermore, twelve studies delved into the expression levels of proteins such as p21-Ras, p38MAPK, p53, Bcl-2, Bax, and others. The detailed characteristics of the included studies are shown in Table 2 and Table 3.

**TABLE 2:** Characteristics of included documents (Animal experiments).

Literature	Objects	Weight	Anaesthesia	Grouping and administration	Result	Mechanism	Difference between groups
Tang and Sun (1986)	C_57_BL, Kunming mice, both	18–22 g	NR	Control group: normal saline	①FC for gastric cancer: ↓ tumor weight	____	①*p* < 0.01 (0.4 mg/mL)
				Aconitine group: 0.2, 0.1, 0.05, 0.4 mg/mL	②↓ tumor weight (sarcoma S_180_)		②*p* < 0.05 (0.2 mg/mL)
				Administration time: 14 d	③spontaneous metastasis of LLC: ↓ tumor weight		③*p* < 0.001 (0.4 mg/mL)
Yang et al. (2005)	C_57_ mice, male (SPF)	18–20 g	NR	Tumor bearing group: distilled water	①↓ tumor weight	____	①*p* < 0.001 (0.80 g/kg)
				Normal group: distilled water	②↓ thymus index		②*p* < 0.01 (0.80 g/kg)
				Aconitine group: 0.51, 0.64, 0.80 mg/kg	③↓ number of metastases		③*p* < 0.001 (0.51, 0.64 g/kg), *p* < 0.01 (0.80 g/kg)
				Administration time: 18 d			
Zeng et al. (2007)	Kunming mice, both (SPF)	18–22 g	Cervical dislocation	Model group: normal saline	①↓ tumor weight	____	①*p* < 0.01 (60 g/kg)
				Aconitine group: 0.15, 0.30, 0.60 mg/mL			
				Administration time: 10 d			
Wu et al. (2018)	ICR mice, both	20 ± 2 g	NR	Control group: distilled water	①↑ mononuclear macrophage phagocytosis	____	①*p >* 0.05 (50, 100, 150 mg/kg)
				Positive drug: Levamisole hydrochloride, 100 mg/kg	② ↑serum IgG level		②*p* < 0.01 (50, 100 mg/kg), *p* < 0.05 (150 mg/kg)
				Aconitine group: 0.50, 1.00, 1.50 mg/kg			
				Administration time: 7 d			
Ren and Zeng (2008)	Kunming mice, male	18–22 g	Cervical dislocation	Blank group: Normal saline, 0.2 mL	⑴↓ tumor weight	⑴↑ TNF-α, ↓ NF-κB, ↑ caspase-3	①*p* < 0.01
				Aconitine group: 2 mg/kg			⑴*p* < 0.05
				Administration time: 10 d			
Zhao et al. (2014)	C57BL mice, male (SPF)	20 ± 2 g	NR	Normal group: normal saline	①↑ 10 min autonomous activity frequency	____	①*p* < 0.01
				Model group: 0.5% CMC-Na	②↑ heart oxygen saturation, ↑ body temperature, ↓ plasma viscosity		②*p* < 0.01 (3 g/kg), *p* < 0.05 (1 g/kg)
				Positive drug: DOX, 5 mg/kg	③↑ erythrocyte ATPase		③*p* < 0.01
				Aconitine group: 1 mg/kg, 3 mg/kg	④↓ intratumoral/pulmonary capillary permeability		④*p* < 0.01
				Administration time: 5 d	⑤↓ tumor/lung HIF-1α, ↓ tumor weight		⑤*p* < 0.01
					⑥↑ intratumoral/pulmonary oxygen saturation		⑥*p* < 0.01
					⑦↓ number of pulmonary metastatic nodules		⑦*p* < 0.01
Ji et al. (2016)	Athymic nude mice, male	20 ± 2 g	NR	Model group: methanol	①↓ tumor volume	____	①*p* < 0.01 (50, 100 mg/kg, 24, 28 d)
				Aconitine group: 0.50, 1.0 mg/kg	②↓ tumor weight		②*p* < 0.01 (50 mg/kg), *p* < 0.001 (100 mg/kg)
				Administration time: 28 d			
Ma (2017)	Kunming mice, male	18–22 g	NR	Model group: 0.5% CMC-Na	①↑ body weight (10 w), → body weight (16 w)	⑴↑ E-Cadherin, ↑ Cytokoratin-18, ↓ N-Cadherin, ↓ Vimentin protein expression, ↓ OCT-4, ↓ NANOG, ↓ PCNA protein expression	①NR
				Aconitine group: 0.2 mg/kg	②↓ 5min autonomous activity		②NR
				Administration time: 26 w	③↓ lung index		③*p* < 0.05
					④↓ number of pulmonary nodules		④*p* < 0.01
							⑴NR
Qi et al. (2018)	C57BL/6J mice, female (SPF)	18–22 g	NR	Model group: normal saline, 5 mL/kg	①↓ tumor volume	⑴→PD-L1 mRNA expression	①*p* < 0.05
				Aconitine group: 36.0 mg/10 g	②↓ tumor weight, ↓ tumor inhibition rate		②*p* < 0.05
				Administration time: 16 d	③↑ apoptosis rate		③*p* < 0.05
					④↑ IL-2, → IL-5, ↑ IL-6, ↑ IL-10, → IL-12, ↓ TGF-β content		④*p* < 0.05 (Il-2, IL-6, IL-10, TGF-β)
					⑤→ Treg ratio of LLC model mice		⑤*p >* 0.05
							⑴*p >* 0.05
Qi et al. (2018)	BALB/c nude mice, male	20 ± 2 g	NR	Control group: PBS	①↓ tumor volume	____	①*p* < 0.01 (2, 4 mg/kg)
				Aconitine group: 2, 4 mg/kg	②↑ survival time of mice		②NR
				Administration time: 21 d			
Wu et al. (2018)	FVB mice, male	20–22 g	Cervical dislocation	Control group: 0.1% DMSO	①↑ ARE fluorescein activity	⑴↑ MRP2, ↑ BCRP protein expression	①*p* < 0.001 (25, 50, 100 μM)
				Aconitine group: 0.6 mg/kg		⑵↑ MRP2, ↑ BCRP gene expression level	⑴*p* < 0.001 (Jejunum, Ileum, Colon)
				Administration time: 14 d		⑶↑ Nrf2/β-actin expression rate	⑵*p* < 0.05 (Jejunum, Ileum, Colon)
						1 ↑ MRP2, ↑ BCRP expression rate	⑶*p* < 0.001 (Jejunum), *p* < 0.01(Colon), *p* < 0.01 (Ileum)
							⑷*p* < 0.01 (Jejunum, Ileum, Colon)
Cheng (2019)	615 mice, both (SPF)	24 ± 2 g	Cervical dislocation	Normal group: sterile normal saline	①↓ tumor volume	____	①*p* < 0.05
				Model group: sterile normal saline	②↓ tumor weight, ↑ tumor inhibition rate		②*p* < 0.01 (0.15 mg/mL)
				Positive drug: Celecoxib, 0.5 mg/mL	③↓ Treg ratio of peripheral mononuclear cells in mice		③*p* < 0.01 (0.1, 0.15 mg/mL)
				Aconitine group: 0.05, 0.1, 0.15 mg/mL	④↓ Treg ratio of mouse spleen mononuclear cells		④*p* < 0.01 (0.15 mg/mL)
				Administration time: 14 d	⑤↓ PGE2 content in peripheral blood of mice		⑤*p* < 0.01 (0.05, 0.1 mg/mL)
					⑥↑ survival time of mice		⑥*p* < 0.05 (0.15 mg/mL)
Wang (2020)	BaLB/c mice, male (SPF)	20 ± 2 g	0.1 mL 1% Pentobarbital solution	Normal group: sterile normal saline 0.2 mL	①↑ weight, ↑ body mass	⑴→ NKp46, ↓ NKG2D, ↓ TIGIT, → TACTILE expression level	①*p* < 0.05 (0.5 mg/mL), *p* < 0.05 (0.5 mg/mL)
				Model group: sterile normal saline 0.2 mL	②↓ growth rate of subcutaneous tumor	⑵↑ CD107a expression	②*p* < 0.05
				Aconitine group: 0.125, 0.25, 0.5 mg/mL, 0.2 mL	③↑ tumor inhibition rate		③*p* < 0.05 (0.25 mg/mL), *p* < 0.01 (0.5 mg/mL)
				Administration time: 21d	④↑ thymus Index		④*p* < 0.05 (0.5 mg/mL)
					⑤↓ spleen index		⑤*p* < 0.05 (0.25 mg/mL), *p* < 0.01 (0.5 mg/mL)
					⑥↑ TNF-α, ↑ IL-1β, ↑ IFN-γ, → B cells, ↑ T cells content		⑥*p* < 0.05 (0.5 mg/mL)
					⑦↑ NK cell number		⑦*p* < 0.05 (0.5 mg/mL)
							⑴*p* < 0.05 (0.5 mg/mL)
							⑵*p* < 0.05 (0.5 mg/mL)

**TABLE 3:** Characteristics of included documents (Cell experiment).

Literature	Cell lines	Grouping and concentration	Results	Mechanism	Difference between groups
Yang et al. (2005)	LM_2_ cell line	Solvent control: RPMI-1640	①↑ 24 h apoptosis rate, ↑ 48 h apoptosis rate	____	①*p* < 0.01 (5, 25 mg/mL, 24, 48 h), *p* < 0.05 (75 mg/mL, 24, 48 h)
		Aconitine group: 5, 25, 75 mg/mL (24, 48 h)			
Zeng et al. (2007)	LoVo, MGC803 cell line	Solvent control: RPMI-1640	①↓ OD value	____	①NR
		Aconitine group: 10, 40, 80, 100, 200, 400, 600, 800 (10^–3^ g/mL)	②↑ inhibition rate		②NR
Xu et al. (2008)	S_180_ cells were derived from Kunming mice	Blank control group: RPMI-1640, cell-free	①↓ absorbance A	____	①NR
		Positive drug: 5-FU, 0.083 mg/mL	②↑ tumor inhibition rate		②*p* < 0.01
		Aconitine group: 0.01, 0.1, 1.0 mg/mL			
Zhu (2008)	AGS, HepG2 and A549 cell line	Negative control: RPMI-1640	①↑ tumor inhibition rate	____	①*p* < 0.05
		Blank control group: normal saline, cell-free			
		Blank control group: 0.05% DMSO, cell-free			
		Positive drug: 5-FU, 100 μg/mL			
		Aconitine group: 0.05, 0.5, 5, 50 μg/mL			
Rao and Peng (2010)	Retinal nerve cells of rats were derived from SD rats and suckling rats	Negative control: 5% CO_2_, 20% DMEM, cell-free	①↓ G_0_/G_1_ phase, ↓ S phase and ↑ G_2_/M phase	⑴↓ p21-Ras gene and protein expression	①*p* < 0.05
		Aconitine group: 0.5%, 1 mL			⑴*p* = 0.036 < 0.05
Zhang and Wu (2011)	SGC-7901, moderately differentiated adenocarcinoma cell line	Blank control group: RPMI-1640, cell-free	①↑ tumor inhibition rate	____	①*p >* 0.05 (20 mg/mL, 24 h), *p* < 0.05 (20 mg/mL, 48, 72 h), *p* < 0.05 (40, 80 mg/mL, 24, 48, 72 h)
		Positive drug: 5-FU, 50 μg/mL	②↑ apoptosis rate		②*p >* 0.05 (40, 80 mg/mL, 24 h)
		Aconitine group: 20, 40, 80 mg/mL (24, 48, 72 h)			
Liu (2013)	Eca-109, MGC80-3, BGC-823, A549, NCI-H460, NCI-H446, MCF-7, SK-OV-3, A375, K-562, U937, S-180 and MHCC97-H cell lines	Positive drug: DDP, 0.15, 0.75, 1.5, 5, 15, 50 μg/mL	①↑ tumor inhibition rate	____	①NR
		Aconitine group: 3, 10, 30, 100, 300, 1000 μg/mL (72 h)			
Ding et al. (2013)	SGC-7901 cell lines	Blank control group: PBS, cell-free	①↑ cell proliferation inhibition rate	____	①*p* < 0.01 (0.05, 0.1, 0.2, 0.4, 0.8 mg/mL, 24, 48, 72 h)
		Aconitine group: 0.05, 0.1, 0.2, 0.4, 0.8 mg/mL (24, 48, 72 h)	②↑ apoptosis rate		②*p* < 0.05 (0.2, 0.4, 0.8 mg/mL)
			③↓ G_0_/G_1_phase, ↓ G_2_/M phase, ↑ S phase		③NR
Ding (2014)	SGC-7901 cell lines	Blank control group: PBS, cell-free	①↑ inhibition rate	____	①*p* < 0.01 (200, 400, 800 μg/mL, 24, 48, 72 h)
		Aconitine group: 50, 100, 200, 400, 800 μg/mL (24, 48, 72 h)	②↓ IC_50_		②NR
			③↑ inhibition rate of cell invasion		③*p* < 0.01 (100, 200, 400 μg/mL)
			④↑ early apoptosis rate		④*p* < 0.01 (100, 200, 400 μg/mL)
			⑤↓ G_0_/G_1_ phase, ↓ G_2_/M phase, ↑ S phase		⑤NR
Hao (2014)	SGC-7901, HepG2 and A549 cell lines	Blank control group: RPMI-1640, cell-free	①↓ OD value of cell proliferation of SGC-7901	____	①*p* < 0.01 (5, 10, 50, 100 μg/mL), *p* < 0.05 (1 μg/mL)
		Aconitine group: 1, 5, 10, 50, 100 μg/mL	②↓ OD value of cell proliferation of HepG2		②*p* < 0.01 (5, 10, 50, 100 μg/mL), *p* < 0.05 (1 μg/mL)
			③↓ OD value of cell proliferation of A549		③*p* < 0.01
Zhao et al. (2014)	Lewis lung cancer cells were derived from C57BL/6 mice	Model group: RPMI-1640, cell-free	①↓ cell proliferation	____	①*p* < 0.01
		Aconitine group: 0.2、0.1、0.05、0.025 μg/mL	②↓ cell adhesion		②*p* < 0.01
			③↑ SDH content		③*p* < 0.01 (0.2, 0.1 μg/mL), *p* < 0.05 (0.05 g/mL)
Fan et al. (2016)	A549 cell lines	Negative control: No drugs	①↑ apoptosis rate	⑴↑ p38 MAPK expression level	①*p* < 0.05
		Aconitine group: 100, 200, 400, 800 μg/mL	②↓ ΔΨm of A549 cell	⑵↑ DRS,↑ TNF-R1 expression level	②NR
				⑶↑ p53, ↑ Bax, ↓ Bcl-2 expression level	⑴*p* < 0.05
				⑷↓ expression of Cytochrome C	⑵*p* < 0.05
				⑸↑ cleaved caspase-9, ↓ pro-caspase-8, ↓ pro-caspase-3 expression level	⑶*p* < 0.05
					⑷*p* < 0.05
					⑸*p* < 0.05
Guan et al. (2015)	K562 cell lines	Blank control group: RPMI-1640, cell-free	①↑ cell proliferation inhibition rate	____	①*p* < 0.05 (25, 50 mg/L, 24 h)
		Solvent control: PBS	②↓ number of G_1_ cells, ↑ number of S cells		②*p* < 0.05 (25, 50 mg/L, 72 h)
		Aconitine group: 5, 10, 25, 50, 75, 100 mg/L (24, 48, 72 h)	③↑ 72 h apoptosis rate		③*p* < 0.05 (25, 50 mg/L, 72 h)
Ji et al. (2016)	Pancreatic cancer cell lines miapaca-2 and PANC-1	Negative control: No drugs	①↑ tumor inhibition rate	⑴↓ NF-κB, ↑ Bax, ↓ Bcl-2, ↑ cleaved caspase-9,↑ cleaved caspase-3, ↑ cleaved PARP, ↑ cyto.C protein level	①*p* < 0.01 (30 μM)
		Aconitine group: 10, 20, 40, 80 μM (24h, 48, 72 h)	②↓ cell colony forming number	⑵↑ caspase-3, → caspase-8, ↑ caspase-9 relative activity	②*p* < 0.05 (15, 30 μM), *p* < 0.01 (60 μM)
			③↑ apoptosis rate		③*p* < 0.05 (30, 60 μM)
					⑴*p* < 0.0.5 (15, 30, 60 μM)
					⑵*p* < 0.05 (15, 30, 60 μM)
Guan et al. (2017)	K562, K562 daunorubicin resistant cell lines	Blank control group: RPMI-1640, cell-free	①↑ K562 cell proliferation inhibition rate	⑴↓ C/EBP-α, ↑ caspase-3, ↑ p53 gene expression	①*p* < 0.05 (10, 25, 50, 75, 100 μmol/L, 24, 48, 72 h)
		Solvent control: PBS	②↑ K562/DNR cell proliferation inhibition rate		②*p* < 0.05 (10, 25, 50, 75, 100 μmol/L, 24, 48, 72 h)
		Aconitine group: 5, 10, 25, 50, 75, 100 μmol/L (24, 48, 72 h)	③↑ K562, K562/DNR apoptosis rate		③*p* < 0.05 (25, 50 μmol/L, 72 h)
					⑴*p* < 0.05 (50 μmol/L, C/EBP-α, Caspase-3, p53)
Ma (2017)	Mouse Lewis lung cancer cells (LLC)	Blank control group: DMEM, cell-free	①↓ LLC cell malignant proliferation fluorescence	⑴↓ Oct-4, ↓ NANOG, ↓ PCNA expression level	①*p* < 0.05
		Aconitine group: 0.1, 1, 10, 100, 1000 μg/mL (72 h)	②↓ LLC cell erosion ability	⑵↑ EGF, ↑ FGF, ↑ HGF, ↑ OSM, ↑ β-actin expression	②*p* < 0.05
			③↓ Cell self-renewal ability		③*p* < 0.05
					⑴*p* < 0.01
					⑵*p* < 0.05
Ma and Yu (2018)	Peripheral blood B lymphocytes of multiple myeloma (RPMI8226)	Blank control group: RPMI-1640, cell-free	①↑ cell proliferation inhibition rate	____	①*p* < 0.05 (4, 6, 8 μmol, 12, 24, 36, 48 h)
		Negative control: PBS and absolute ethanol	②↑ apoptosis rate		②*p* < 0.05 (4, 8 μmol, 24, 48 h)
		Aconitine group: 0.1, 0.5, 1, 10, 100 μmol/mL (24, 48 h)			
Qi et al. (2018)	HepG2, Huh7 and L02 cells	Negative control: No drugs	①↓ cell viability	⑴↑ Bax, ↓ Bcl-2, ↑ cleaved caspases-3, ↑ cleaved caspases-7, ↑ cleaved caspases-PARP, → GAPDH protein expression	①*p* < 0.05 (6.25, 12.5 μg/mL, 24 h), *p* < 0.01 (6.25, 12.5, 25, 50, 100 μg/mL, 48,72 h)
		Aconitine group: 6.25, 12.5, 25, 50, 100 μg/mL (24, 48, 72 h)	②↑ apoptosis rate	⑵↑ cytochrome c/tublin, ↓ cytochrome c/Cox IV content	②*p* < 0.01 (25, 50 μg/mL)
			③↑ ROS content		③*p* < 0.01 (25, 50 μg/mL)
					⑴*p* < 0.01 (50 μg/mL), *p* < 0.05 (25 μg/mL)
					⑵*p* < 0.01 (25, 50 μg/mL)
Wu et al. (2018)	human colon cancer cell line LS174T, Caco-2	Blank control group: DMSO	①↓ accumulation of CDF	⑴↑ MRP2, ↑ BCRP protein level	①*p* < 0.01
		Aconitine group: 5, 10, 20 μM (6,12 h)		⑵↑ MRP2, ↑ BCRP expression rate	⑴*p* < 0.001 (Jejunum, Ileum, Colon)
				⑶↑ MRP2/GAPDH, ↑ BCRP/GAPDH expression rate	⑵*p* < 0.01 (Colon), *p* < 0.05 (Jejunum, Ileum)
				⑷↑ MRP2, BCRP immunofluorescence rate	⑶*p* < 0.01 (5, 10, 20 μM)
					⑷*p* < 0.05 (20 μM)
Xiong et al. (2018)	Human hepatoma cell line MHCC97	Blank control group: DMEM, cell-freeAconitine group: 5, 10, 20 μg/mL (96 h)	①↓ proliferation number of hepatoma cells	⑴↓ pP38, ↓ P38, ↓ p-MAPKAPK, ↓ p-HSP27 signal pathway expression	①*p* < 0.05 (10, 20 μg/mL)
			②↓ invasiveness of hepatoma cells		②*p* < 0.05 (10, 20 μg/mL)
			③↓ migration ability of hepatoma cells		③*p* < 0.05 (5, 10, 20 μg/mL)
					⑴*p* < 0.05 (5, 10, 20 μg/mL)
Zhang et al. (2018)	H9c2 cell lines	Blank control group: DMSO, cell-free	①↑ inhibition rate to H9c2	____	①*p* < 0.05
		Aconitine group: 150, 250, 400, 500, 1000 μg/mL (24 h)	②↑ LDH leakage rate		②*p* < 0.05 (100, 400, 500 μg/mL)
			③↑ apoptosis rate		③*p* < 0.05 (400, 500 μg/mL)
Shao et al. (2019)	A549 cell lines	Blank control group: RPMI-1640, cell-free	①↑ cell proliferation inhibition rate	⑴↓ Bcl-2, ↑ Bax, ↑ caspase-3, ↑ Beclin1, ↑ LC3, ↓ P62 mRNA expression level	①*p* < 0.05 (100,200 μmol/L), *p* < 0.01 (400 μmol/L)
		Aconitine group: 10, 50, 100, 200, 400 μmol/mL (24,48,72 h)	②↑ 48 h apoptosis rate	⑵↓ Bcl-2, ↑ Bax, ↑ active caspase-3, ↑ Beclin1, ↑ LC3 II/I, ↑ P-62 protein expression level	②*p* < 0.01 (200, 400 μmol/L)
					⑴*p* < 0.05 (Beclin1, LC3 II/I, P-62, 400 μmol/L), *p* < 0.01 (Bcl-2, Bax, Caspase-3, 400 μmol/L)
					⑵*p* < 0.05 (Bcl-2, Bax, 400 μmol/L), *p* < 0.01 (Active caspase-3, Beclin1, LC3 II/I, P-62, 400 μmol/L)
Zhou et al. (2019)	Human esophageal carcinoma EC-1 cells	Blank control group: DMEM, cell-free	①↑ tumor inhibition rate	⑴↓ MMP-9, ↓ Bcl-2 protein expression level	①*p* < 0.05
		Negative control: No drugs	②↓ cell clonogenic ability		②*p* < 0.05, *F* = 127.59 (6.25, 12.5 μg/mL)
		Aconitine group: 0.8, 1.6, 3.2, 6.25, 12.5, 25.0 μg/mL (24,48,72 h)	③↓ cell invasiveness		③*p* < 0.05, *F* = 204.34 (6.25, 12.5 μg/mL)
			④↑ apoptosis index		④*p* < 0.05, *F* = 428.56 (6.25, 12.5 μg/mL)
					⑴*p* < 0.01 (6.25, 12.5 μg/mL)
Cheng et al. (2020)	Mouse peripheral blood mononuclear cells were from 615 mice (SPF grade)	Negative control: RPMI-1640, cell-free	①↓ PGE2 content in monocytes of 615 mice	____	①*p* < 0.05 (2 mg/mL)
		Positive drug: Celecoxib, 2.5 μg/mL	②↓ percentage of Tregs differentiated from monocytes in 615 mice		②*p* < 0.05 (0.5 mg/mL), *p* < 0.01 (0.1, 2 mg/mL)
		Aconitine group: 0.1, 0.5, 2 mg/mL			
Jia and Zhang (2011)	Human gastric adenocarcinoma SGC-7901 cell line was derived from SD rats	Control group: normal saline	①↓ SGC-7901 cell proliferation	____	①*p* < 0.05
		Positive drug: 5-FU, 250 mg/L			
		Aconitine group: 1:1000 (24,48,72 h)			
Ru et al. (2020)	Gastric adenocarcinoma cell line MGC803	Solvent control: DMEM, cell-free	①↓ proliferation of gastric adenocarcinoma cells	⑴↑ miR-23a expression, ↑ IRF1 gene expression level	①*p* < 0.05
		Negative control: No drugs	②↓ cell colony forming ability		②*p* < 0.05 (40 μg/mL)
		Aconitine group: 5, 10, 20, 40, 60, 80, 100 μg/mL (24,48,72 h)	③↑ apoptosis index		③*p* < 0.05 (40 μg/mL)
					⑴*p* < 0.05 (40 μg/mL, miR-23a), *p* < 0.01 (40 μg/mL, IRF1)
Wang (2020)	Human hepatoma cell lines Huh-7, MHCC-97h, MHCC -lm3, BEL-7402, Hep-G2, Hep -3B, SMMC-7721	Blank control group: DMEM+10% FBS, cell-free	①↓ OD value of hepatoma cells (48, 72 h)	____	①*p* < 0.05
		Negative control: No drugs	②↑ apoptosis rate of hepatocellular carcinoma cells		②*p* < 0.01 (high dose)
		Aconitine group: 250, 500, 1000, 2000, 4,000, 8,000 μg/mL (24,48,72 h)	③↓ migration ability of hepatoma cells		③NR
Wang et al. (2020)	Human oVca cell lines, a2780 and normal ovarian cell ioSe80	Negative control: No drugs	①↓ cell viability	⑴↑ ERβ, ↓ VEFG expression level	①*p* < 0.01 (50, 100, 200, 400, 800, 1000 μg/mL, 24 h)
		Positive drug: DDP, 1, 5, 25, 50, 100 μg/mL	②↓ cell colony forming number, ↓ cell invasiveness, ↓ cell migration ability	⑵↓ HiF-α, ↑ PHd2, ↓ MMP2, ↓ MMP9, ↓ aTM, ↓ p-aTM, ↑ p53 expression level	②*p* < 0.01 (100, 200, 400 μg/mL), *p* < 0.01 (25, 50, 100 μg/mL), *p* < 0.01 (100 μg/mL)
		Aconitine group: 10, 50, 100, 200, 400, 800, 1000 μg/mL (6,12,24 h)	③↑ ΔΨm	⑶↑ Bax, ↓ Bcl-2, ↑ apaf-1, ↑ cleaved caspase-3, ↑ cleaved caspase-9, ↓ Bcl-xl, ↑ Cyt C, ↑ cleaved ParP protein expression level	③*p* < 0.01 (400 μg/mL), *p* < 0.05 (100, 200 μg/mL)
			④↑ apoptosis rate		④*p* < 0.05 (100, 200, 400 μg/mL)
					⑴*p* < 0.05 (100 μg/mL), *p* < 0.01 (200, 400 μg/mL)
					⑵*p* < 0.01 (200, 400 μg/mL)
					⑶*p* < 0.01 (200, 400 μg/mL)
Zhang et al. (2020)	A549 human lung cancer cell line	Negative control: No drugs	①↑ A549 cell proliferation inhibition rate	____	①*p* < 0.01
		Positive drug: DDP, 0.5, 1, 5, 4, 6 μg/mL	②A549 cell growth curve		②NR
		Aconitine group: 300, 600, 900, 1200, 1500 μg/mL (24,48,72 h)			
Zou et al. (2021)	Human osteosarcoma 143B cells	Negative control: No drugs	①↓ cell viability	⑴↑ caspase-3, ↑caspase-9	①*p* < 0.05
		Aconitine group: 3.125, 6.25, 12.5, 25.00, 50.00 μg/mL (12,24,36 h)	②↑ ROS positive rate		②*p* < 0.05 (3 μmol/L)
			③↑ Gray scale ratio (p-JNK/β- actin)		③*p* < 0.05 (3 μmol/L)
			④↑ apoptosis rate		④*p* < 0.05 (3 μmol/L)
			⑤caspase mediated apoptosis		⑤NR
					⑴*p* < 0.05 (3 μmol/L)
Cai (2022)	Prostate cancer cell DU145	Negative control: No drugs	①↓ cell proliferation and survival rate	⑴↑ Bax, ↓ Bcl-2, ↓ p-JAK2, → JAK2, → p-STAT3, → STAT3 protein level	①*p* < 0.05 (10,20 μg/mL)
		Aconitine group: 5, 10, 20 μg/mL (48 h)	②↓ number of cell invasion		②*p* = 0.000 < 0.05, t = 13.702
			③↑ apoptosis rate		③*p* = 0.000 < 0.05, t = 29.336
					⑴*p* < 0.05 (Bax, Bcl-2), *p* < 0.05 (p-JAK2, p-STAT3), *p >* 0.05 (JAK2, STAT3)
Luan et al. (2022)	MCF-7, MCF-7/ADR, NIH3T3 cell line	Positive drug: DOX, etoposide, 0.2 mL	①↑ anti-increment activity	____	①*p* < 0.05
		Aconitine group: 5, 10, 15 μM (48 h)	②↑ G_0_/G_1_ phase, ↓ S phase		②NR

Note, NR, not report; ↑, Upregulated expression, rising and increasing; ↓, Downregulated expression, decreased; →, Unchanged; DDP, cisplatin; 5-Fu, 5-Fluorouracil; DOX, doxorubicin; SDH, succinate dehydrogenase; ΔΨm, Mitochondrial membrane potential; p38MAPK, P38 mitogen activated protein kinase; TNF-R1, tumor necrosis factor-R1; Bax, Bcl-2-associated X protein; Bcl-2, B cell lymphoma 2; Cyt C, Cytochrome C; C/EBPα, regulatory transcription factors; EGF, epidermal growth factor; FGF, fibroblast growth factor; HGF, hepatocyte growth factor; OSM, oncostatin-M; LDH, lactate dehydrogenase; Beclin1, Autophagy effector protein 1; MMP, matrix metalloproteinase; PGE2, Prostaglandin e2; IRF1, Interferon regulatory factor 1; Erβ, estrogen receptor β; VEGF, vascular endothelial growth factor A; HiF-α, hypoxia-inducible factor; PHd2, prolyl hydroxylase domain-containing protein 2; aTM, aTM, serine/threonine kinase; p-, phosphorylated; apaf-1, apoptotic peptidase activating factor 1; ParP, poly (adP-ribose) polymerase; PARP, poly (ADP-ribose) polymerase; Z-VAD-FMK, benzyloxycarbonyl-Val-Ala-Asp-fluoromethylketone; ROS, reactive oxygen species; COX IV, cytochrome oxidase subunit IV; E-Cadherin, Epithelial cadherin; PCNA, proliferating cell nuclear antigen; MRP2, multi-drug resistance protein 2; BCRP, breast cancer resistance protein; NKp46, NK, cell protein 46.

### 3.3 Included in literature quality evaluation

The CAMARADES evaluation form was utilized to assess the quality of the literature included in the 37 preclinical trials, as presented in Table 4. The studies were evaluated on a scale of 4-7, with an average score of 5.32. Among these studies, 4 (Zhao et al., 2014; Zhang et al., 2017; Wu et al., 2018; Cheng et al., 2020); 10 (Zeng et al., 2007; Rao and Peng, 2010; Liu, 2013; Ji et al., 2016; Ma, 2017; Qi et al., 2018b; Cheng, 2019; Wang, 2020; Wang et al., 2020; Luan et al., 2022) studies received 6 points; 18 (Xu et al., 2008; Zhu, 2008; Jia and Zhang, 2011; Zhang and Wu, 2011; Ding et al., 2013; Ding, 2014; Guan et al., 2015; Fan et al., 2016; Guan et al., 2017; Ma and Yu, 2018; Xiong et al., 2018; Zhang et al., 2018; Shao et al., 2019; Zhou et al., 2019; Ru et al., 2020; Zhang et al., 2020; Zou et al., 2021; Cai, 2022) studies received 5 points; 5 (Tang and Sun, 1986; Yang et al., 2005; Ren and Zeng, 2008; Wuliji et al., 2008; Hao, 2014) studies received 4 scores. It is worth noting that all of these studies went through the peer review process before publication. While all studies were randomized, 11 of them (Tang and Sun, 1986; Yang et al., 2005; Zeng et al., 2007; Ren and Zeng, 2008; Wuliji et al., 2008; Rao and Peng, 2010; Hao, 2014; Zhang et al., 2017; Wu et al., 2018; Cheng, 2019; Wang, 2020) failed to provide an accurate description of their grouping method, and 3 (Tang and Sun, 1986; Yang et al., 2005; Ren and Zeng, 2008) studies did not mention temperature control. Additionally, none of the studies mentioned implementing model-blinded or outcome-blinded methods. 9 (Zeng et al., 2007; Ren and Zeng, 2008; Rao and Peng, 2010; Zhao et al., 2014; Zhang et al., 2017; Wu et al., 2018; Cheng, 2019; Cheng et al., 2020; Wang, 2020) studies were found to mention lethal mode or anesthetic use. All of these studies utilized appropriate animal or cellular models and calculated the necessary data. 12 (Tang and Sun, 1986; Yang et al., 2005; Zeng et al., 2007; Rao and Peng, 2010; Liu, 2013; Zhao et al., 2014; Ma, 2017; Zhang et al., 2017; Wu et al., 2018; Cheng, 2019; Cheng et al., 2020; Wang, 2020) studies mentioned animal welfare regulations, and only 6 (Ji et al., 2016; Zhang et al., 2017; Qi et al., 2018b; Wu et al., 2018; Wang et al., 2020; Luan et al., 2022) studies included a statement regarding potential conflicts of interest.

**TABLE 4:** Quality assessment of the experiments included in the studies.

Study (year)	1	2	3	4	5	6	7	8	9	10	Total
Tang and Sun (1986)	UK	NR	NR	NR	NR	+	+	+	NR	+	4
Yang et al. (2005)	UK	NR	NR	NR	NR	+	+	+	NR	+	4
Zeng et al. (2007)	UK	+	NR	NR	+	+	+	+	NR	+	6
Ren and Zeng (2008)	UK	NR	NR	NR	+	+	+	NR	NR	+	4
Wu et al. (2018)	UK	+	NR	NR	NR	+	+	NR	NR	+	4
Xu et al. (2008)	+	+	NR	NR	NR	+	+	NR	NR	+	5
Zhu (2008)	+	+	NR	NR	NR	+	+	NR	NR	+	5
Rao and Peng (2010)	UK	+	NR	NR	+	+	+	+	NR	+	6
Zhang and Wu (2011)	+	+	NR	NR	NR	+	+	NR	NR	+	5
Ding et al. (2013)	+	+	NR	NR	NR	+	+	NR	NR	+	5
Liu (2013)	+	+	NR	NR	NR	+	+	+	NR	+	6
Ding (2014)	+	+	NR	NR	NR	+	+	NR	NR	+	5
Hao (2014)	UK	+	NR	NR	NR	+	+	NR	NR	+	4
Zhao et al. (2014)	+	+	NR	NR	+	+	+	+	NR	+	7
Fan et al. (2016)	+	+	NR	NR	NR	+	+	NR	NR	+	5
Guan et al. (2015)	+	+	NR	NR	NR	+	+	NR	NR	+	5
Ji et al. (2016)	+	+	NR	NR	NR	+	+	NR	+	+	6
Qi et al. (2018)	UK	+	NR	NR	+	+	+	+	+	+	7
Guan et al. (2017)	+	+	NR	NR	NR	+	+	NR	NR	+	5
Ma (2017)	+	+	NR	NR	NR	+	+	+	NR	+	6
Qi et al. (2018)	+	+	NR	NR	NR	+	+	NR	+	+	6
Wu et al. (2018)	UK	+	NR	NR	+	+	+	+	+	+	7
Ma and Yu (2018)	+	+	NR	NR	NR	+	+	NR	NR	+	5
Xiong et al. (2018)	+	+	NR	NR	NR	+	+	NR	NR	+	5
Zhang et al. (2018)	+	+	NR	NR	NR	+	+	NR	NR	+	5
Cheng (2019)	UK	+	NR	NR	+	+	+	+	NR	+	6
Shao et al. (2019)	+	+	NR	NR	NR	+	+	NR	NR	+	5
Zhou et al. (2019)	+	+	NR	NR	NR	+	+	NR	NR	+	5
Cheng et al. (2020)	+	+	NR	NR	+	+	+	+	NR	+	7
Ru et al. (2020)	+	+	NR	NR	NR	+	+	NR	NR	+	5
Wang et al. (2020)	+	+	NR	NR	NR	+	+	NR	+	+	6
Wang (2020)	UK	+	NR	NR	+	+	+	+	NR	+	6
Zhang et al. (2020)	+	+	NR	NR	NR	+	+	NR	NR	+	5
Jia and Zhang (2011)	+	+	NR	NR	NR	+	+	NR	NR	+	5
Zou et al. (2021)	+	+	NR	NR	NR	+	+	NR	NR	+	5
Cai (2022)	+	+	NR	NR	NR	+	+	NR	NR	+	5
Luan et al. (2022)	+	+	NR	NR	NR	+	+	NR	+	+	6

Note, 1, Random grouping; 2, A statement describing temperature control; 3, Model blind method; 4, Results were evaluated by blind method; 5, The use of anesthetics has no obvious intrinsic myocardial protection or neuroprotective effect; 6, Appropriate animal or cell models; 7, Calculation of sample size; 8, Follow animal welfare regulations; 9, Declare any potential conflict of interest; 10, The paper was published after peer review; UK, unknow, only the random grouping is explained, and the random grouping method is not specified; NR, not report.

### 3.4 Meta analysis results

#### 3.4.1 Tumor weight

**FIGURE 4:**
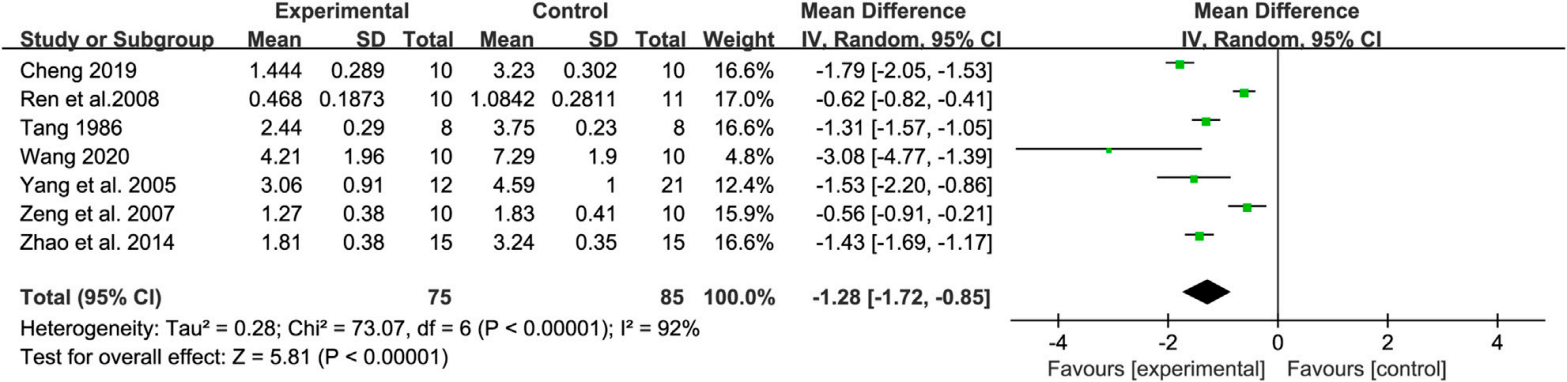
Forest map of tumor weight.

Seven studies were analyzed to compare the change in tumor weight between the control and experimental groups. Heterogeneity was observed between the (*Tau* ^2^ = 0.28, *Chi* ^2^ = 73.07, *df* = 6, *I* ^2^ = 92%, *p* < 0.00001), so a random-effects model was utilized. The findings indicated a significant difference in tumor weight between the treatment and control groups (*Z* = 5.81, *p* < 0.00001) with a 95% confidence interval of [MD = −1.28, 95% CI (−1.72, −0.85)]. The results were shown in Figure 4.

#### 3.4.2 Proliferation number of tumor cells

Cell proliferation is a crucial aspect of an organism’s life, occurring through cell division. Protozoans generate new individuals through cell division, while multicellular organisms produce new cells to replace aging or dying cells within the body (Ji, 2021). Cancer cells, on the other hand, possess three significant traits: infinite proliferation (Jarrett et al., 2018), the ability to transform and metastasize, and the capacity to destroy normal cellular tissues. Therefore, the value-added number of tumor cells is one of the important indicators for the evaluation of antitumor efficacy (Kotel'Nikov, 1989).

**FIGURE 5:**
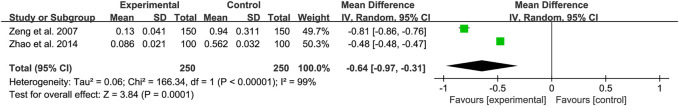
Forest map of proliferation number of tumor cells.

Two studies were analyzed to compare changes in cell proliferation numbers between control and experimental groups. Heterogeneity was observed between trials (*Tau* ^2^ = 0.06, *Chi* ^2^ = 166.34, *df* = 1, *I* ^2^ = 99%, *p* < 0.00001), requiring the use of a random effects model. The results indicated a significant difference between the control and experimental groups (*Z* = 3.84, *p* = 0.0001) with a 95% confidence interval of [MD = −0.64, 95% CI (−0.97, −0.31)]. This indicated that aconitine could reduce the number of tumor cell proliferation in mice. The results were shown in Figure 5.

#### 3.4.3 Thymus index

The thymic index, also referred to as the thymic secretion index, is primarily based on the level of lymphocyte multiplication and can serve as a means to gauge immune intensity (Kubatka et al., 2019).

**FIGURE 6:**
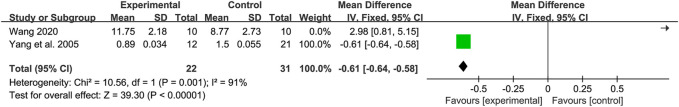
Forest map of thymus index.

A total of two studies were included to compare the change in thymic index between the control and experimental groups. Due to heterogeneity between trials (*Chi* ^2^ = 10.56, *df* = 1, *I* ^2^ = 91%, *p* = 0.001), a random-effects model was utilized. The results showed a statistically significant difference in thymic index between the treatment and control groups (*Z* = 39.30, *p* < 0.00001), with a 95% confidence interval of [MD = −0.61, 95% CI (−0.64, −0.58)]. The results of this study showed that aconitine could reduce the thymic index in mice. The results were shown in Figure 6.

#### 3.4.4 Number of metastatic lesions

Metastatic lesions refer to tumor cells that spread from the primary site through lymphatic vessels, blood vessels, or other pathways to other areas of the body where they continue to grow, forming the same type of tumor as the primary site. This process is known as metastasis, and the resulting tumors are called metastases or metastatic cancer (Suhail et al., 2019). The evaluation of the number of metastatic lesions is frequently used in antitumor studies because of the susceptibility of tumor cells to metastasize.

**FIGURE 7:**
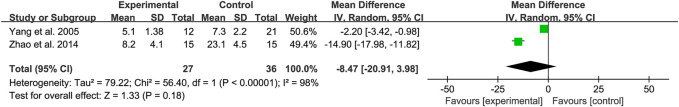
Forest map of number of metastatic lesions.

A total of two studies were included to compare the changes in the number of metastatic lesions between the control and experimental groups, and there was heterogeneity between the trials (*Tau* ^2^ = 79.22, *Chi* ^2^ = 56.40, *df* = 1, *I* ^2^ = 98%, *p* < 0.00001), so a random-effects model was used. T The results indicated that there was no significant difference in the number of metastatic lesions between the treatment and control groups (*Z* = 1.33, *p* = 0.18). Therefore, it cannot be concluded that aconitine has the ability to reduce the number of metastatic lesions in mice. The results were shown in Figure 7.

#### 3.4.5 Tumor cell apoptosis rate

Apoptosis is a genetically controlled process of autonomous and orderly cell death that helps maintain the stability of the internal environment (Elmore, 2007). In contrast, cancer cells can evade apoptosis and grow uncontrollably. As a result, the effectiveness of drugs in treating tumors is frequently evaluated by measuring the apoptosis rate of cancer cells.

**FIGURE 8:**
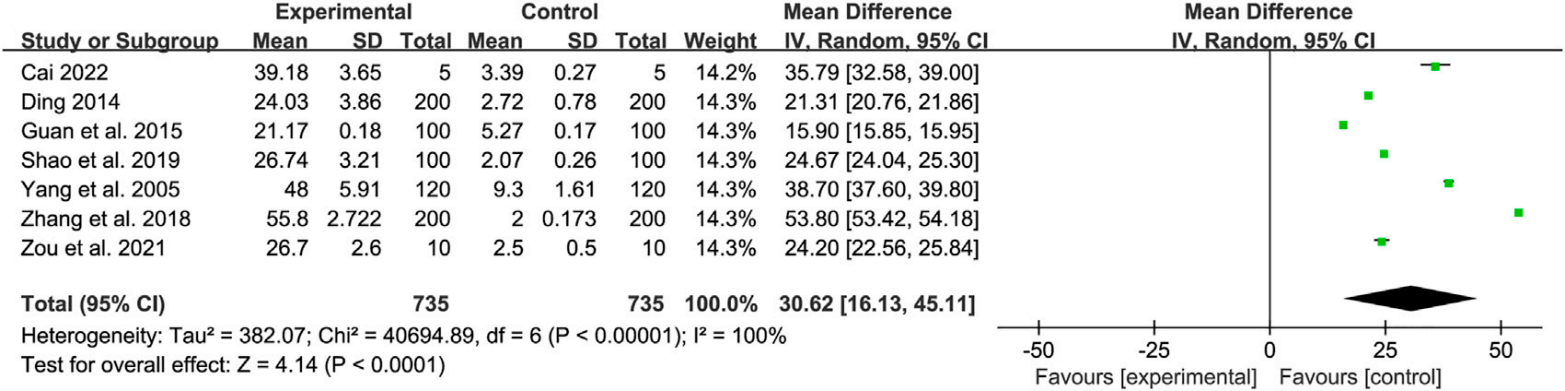
Forest map of tumor cell apoptosis rate.

A total of seven studies were included to compare apoptosis rates between control and experimental groups, and there was heterogeneity between trials (*Tau* ^2^ = 382.07, *Chi* ^2^ = 40,694.89, *df* = 6, *I* ^2^ = 100%, *p* < 0.00001), so a random-effects model was used. The results indicated a significant difference in apoptosis rate between the treatment and control groups (*Z* = 4.14, *p* < 0.0001) with a 95% confidence interval of [MD = 30.62,95% CI (16.13, 45.11)]. The results of this study showed that aconitine could increase the apoptosis rate of tumor cells. The results were shown in Figure 8.

#### 3.4.6 OD value of cell proliferation

The OD value, or optical density value, is a widely used indicator in cellular experiments that measures the amount of light absorbed by the assay. As the number of bacteria in the medium increases, so does the absorbance value within a certain range. Therefore, the OD value is often utilized as a test indicator to assess the proliferation of cells following administration.

**FIGURE 9:**
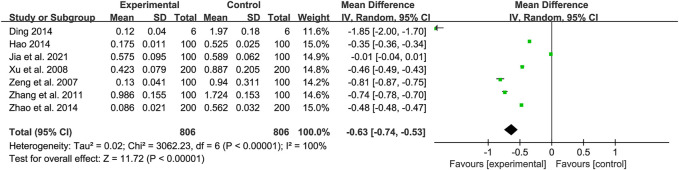
Forest map of OD value of cell proliferation.

A total of seven studies were included to compare the cell proliferation OD between control and experimental groups, with heterogeneity between trials (*Tau* ^2^ = 0.02, *Chi* ^2^ = 3062.23, *df* = 6, *I* ^2^ = 100%, *p* < 0.00001), so a random effects model was used. The results showed a statistically significant difference between the treatment and control groups (*Z* = 11.72, *p* < 0.00001) with a 95% confidence interval of [MD = −0.63, 95% CI (−0.74, −0.53)]. The results of this study showed a decrease in cell proliferation OD. The results were shown in Figure 9.

#### 3.4.7 Bcl-2 expression level

Bcl-2, also known as the B cell lymphoma/leukemia-2, is an oncogene that plays a crucial role in inhibiting cell apoptosis (Ebrahim et al., 2016). As a major target molecule in the study of molecular mechanisms of apoptosis (Bruckheimer et al., 1998), it has garnered significant attention in antitumor research.

**FIGURE 10:**
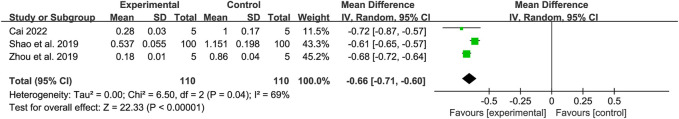
Forest map of Bcl-2 expression level.

A total of three studies were included to compare the anti-cycloguanine peptides between the control and experimental groups. There was some heterogeneity between the trials (*Tau* ^2^ = 0.00, *Chi* ^2^ = 6.50, *df* = 2, *I* ^2^ = 69%, *p* = 0.04), so a random effects model was used. The results revealed a statistically significant difference between the treatment and control groups (*Z* = 22.33, *p* < 0.00001) with a 95% confidence interval of [MD = −0.66, 95% CI (−0.71, −0.60)]. The results of this study showed that aconitine reduced the expression of Bcl-2. The results were shown in Figure 10.

Overall, aconitine demonstrated a more significant anti-tumor effect by reducing tumor weight, inhibiting proliferation and migration of tumor cells, promoting apoptosis, and controlling gene/protein expression of cancer cells, including Bcl-2, Bax, Caspase-3, Caspase-8, Cleaved Caspase-9, and others.

When the value of *I* ^2^ exceeds 50%, it is necessary to conduct a subgroup analysis to investigate the underlying cause of the high heterogeneity. However, the meta-analysis indexes mentioned above have insufficient literature included, such as cell proliferation, thymus index, and number of metastatic lesions indexes. As a result, these indexes may not provide sufficient evidence to support the effectiveness of aconitine against tumors. After reviewing the literature, it became evident that the lack of inclusion of certain studies was because they did not consider the co-administration of aconitine during the screening process.

For instance, one research of Li et al. on the synergistic induction of apoptosis in cervical cancer through the use of quercetin and aconitine, where HeLa cell proliferation was utilized as an outcome indicator (Li et al., 2018); In their study on anti-breast cancer MDA-MB-231BO cells, Guo et al. utilized a combination of osteopontin and aconitine to effectively inhibit cancer cell invasion (Guo et al., 2011); Similarly, Yao et al. found success in treating hepatocellular carcinoma by combining aconitine with Crude Monkshood Polysaccharide, as evidenced by positive experimental outcome indicators such as thymic index (Yao et al., 2021).

The above experiments showed that the meta-analysis indexes used in this study were able to reflect the anti-tumor efficacy of aconitine to some extent. However, due to the absence of quantitative data analysis for integrated and multidimensional purposes in many studies, there is a need for larger sample sizes in *in vivo* or *in vitro* experiments to fully understand the multifaceted antitumor effects of aconitine. This is necessary for a rational and proper assessment of its antitumor efficacy.

## 4 Discussion

### 4.1 Limitation

When conducting a systematic and comprehensive meta-analysis to evaluate the antitumor efficacy of aconitine, it is important to acknowledge certain limitations. First of all, this study only included literature in Chinese and English, which may introduce a selection bias as there may be relevant studies published in other languages that were not considered. Secondly, the various articles investigating the action of aconitine on tumor cells utilize different methods of administration, control groups, doses, and durations of action. As a result, discrepancies arise in the assays and the results become incomparable. Thirdly, a quality assessment score below 5 indicates a low quality of the methods utilized in the study. Furthermore, many of the studies lacked proper reporting and had flaws in their random assignment and blinding of results. These issues make it challenging to assess the studies’ quality and the trustworthiness of the data.

In addition, the majority of the articles lacked raw data and only included analytical plots and corresponding *p* values for comparisons between control groups. This limited our ability to refine the data and analyze the anti-tumor efficacy of aconitine using multiple indicators through meta-analysis. Therefore, it is important to assess and interpret the antitumor effects of aconitine at various levels with rationality. In order to conduct a thorough follow-up protocol on the antitumor efficacy study of aconitine, researchers should consider using a similar or equivalent dose range, frequency, duration of action, and control group during the experimental implementation phase. This will enable them to analyze and study the data more accurately in subsequent in-depth studies.

### 4.2 Implication

The antitumor effects of Aconitine are a complex process that involves multiple factors that have yet to be fully explained. Currently, the main mechanisms of Aconitine anti-tumor properties have been reported in the literature, with a primary focus on the expression of Bax, Bcl-2, Caspase-3, and other proteins, reactive oxygen species damage and the triggering of apoptosis and autophagy in tumor cells.

**FIGURE 11:**
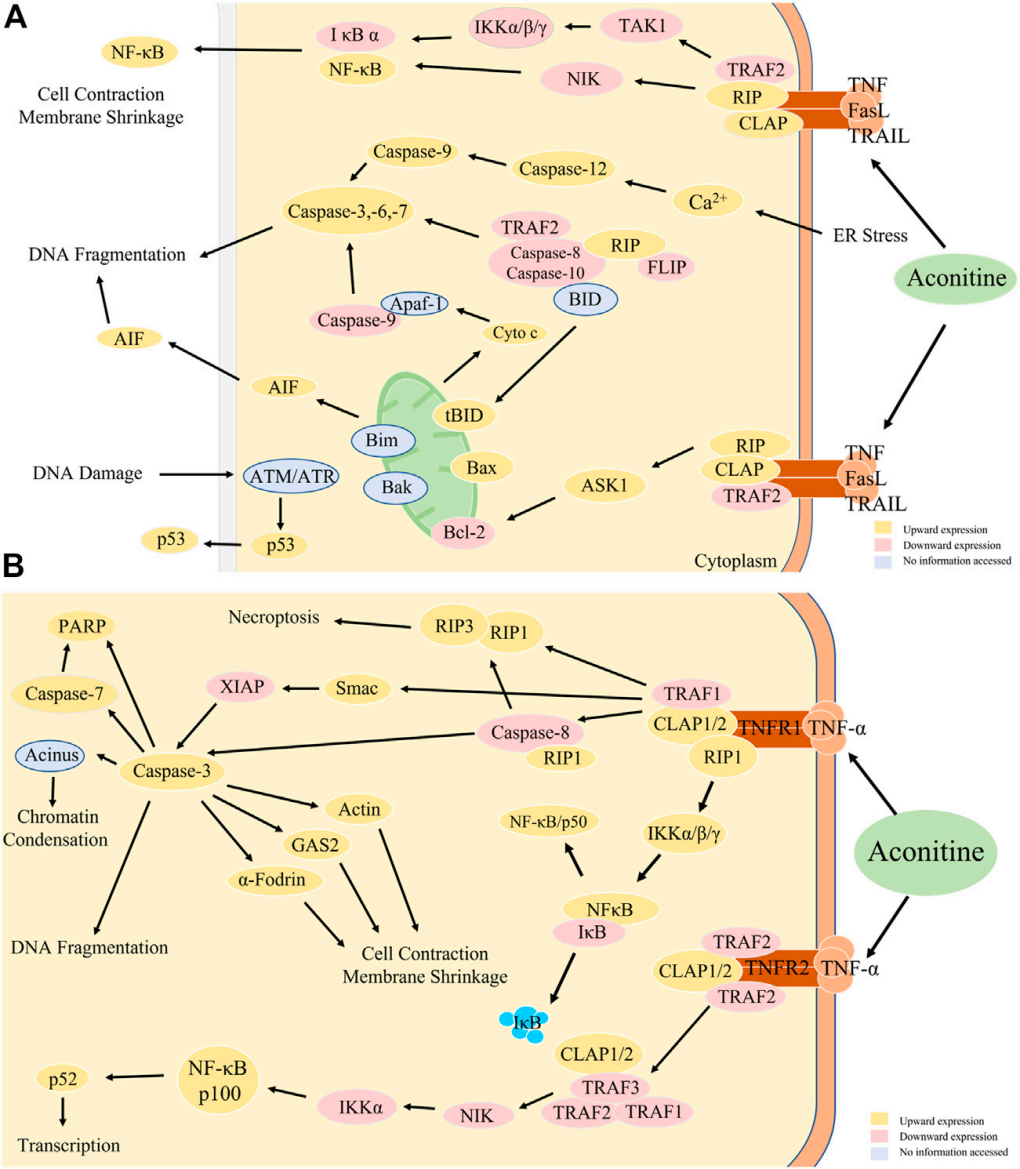
**(A)** Anti-tumor mechanism of aconitine. **(B)** NF-κB signal path diagram. TRAF2, TNF receptor-associated factor 2; NIK, NF-κB-inducible kinase; TAK1, human transforming growth factor kinase 1. IKK, inhibitor of kappa B kinase, kappa B inhibitory factor kinase; FLIP, FLICE inhibitory protein, apoptosis inhibitory protein; Itch (AIP4), atrophin 1 interacting protein 4; RIP, RNA binding protein; ASK1, apoptosis signal-regulated kinase 1; JNK, c-Jun amino-terminal kinase; AIF, apoptosis-inducing factor; BID, carboxy-terminal fragment; ATM, Ataxia-telangiectasia mutated proteins; ATR, Automatic Target Recognition; p53, human tumor suppressor gene; CLAP, Carbohydrates, Lipid, Nucleic Acid, Protein; IκB, inhibitor of NF-κB; RelA, RELA proto-oncogene, NF-κB subunit; Smac, second mitochondria-derived activator of caspases; GAS2, Recombinant Growth Arrest Specific Protein 2; a-Fodrin, Anti-alpha-cytosolic protein; XIAP, X-linked inhibitor of apoptosis protein; PARP, poly ADP-ribose polymerase.

The literature included in this study commonly used animal indicators such as tumor weight, tumor volume, and thymus index. Cellular experiments frequently measured cell proliferation inhibition rate, apoptosis rate, and cell count at different time periods. In mechanism studies, apoptosis-related targets such as Bax, cyto. C, and caspase-3/9 expression were upregulated, while Bcl-2, pro-caspase 9, MMP2/9, and VEGF expression were downregulated. Based on the changes observed in the expression of these targets, we have formulated a hypothesis that suggests the primary signaling pathway for aconitine anti-tumor effects is NF-κB, and the primary acting immune organ is the thymus. The relevant mechanisms were shown in Figure 11A. The mechanisms through which aconitine induces apoptosis in tumor cells, as discussed earlier, offer numerous opportunities for future researchers to delve deeper into the properties of aconitine alkaloids. Additionally, these tests are acknowledged as essential in the development and clinical application of proprietary Chinese medicines that contain aconite medicinal plants.

### 4.3 Thymus immune organs and T lymphocytes

The thymus, a central immune organ, is situated behind the sternum and in close proximity to the heart. It is divided into two lobes, left and right, and primarily composed of the cortex and the medulla (Rodewald, 2008; Albano et al., 2019). The cortex is mainly composed of larger, immature T lymphocytes, while the medulla contains smaller, less abundant mature T lymphocytes. Due to its significance in the immune system, the thymus is frequently studied in antitumor animal model research.

Cytotoxic T lymphocytes (CTL) are a specialized type of T cell that secretes various immune-related cytokines. Along with natural killer cells, they form a crucial line of defense for the body’s antiviral and antitumor immunity (Rascio et al., 2021). CD8^+^ T cells and CD4^+^ T cells are both types of CTL and are believed to have a dominant role in producing an effective anti-tumor response (Kishton et al., 2017).

Cytotoxic T lymphocytes (CTL) are a specialized type of T cell that secretes various immune-related cytokines. Along with natural killer cells, they form a crucial line of defense for the body’s antiviral and antitumor immunity (Rascio et al., 2021). CD8^+^ T cells and CD4^+^ T cells are both types of CTL and are believed to have a dominant role in producing an effective anti-tumor response (Kishton et al., 2017).

CD8^+^ T cells are known to have the ability to directly eliminate tumor cells, making them the preferred immune cells for targeting tumors (Farhood et al., 2019). However, in order to achieve effective anti-tumor immunity, the complementary CD4^+^ T cells are also necessary (Ahrends et al., 2017).

CD8^+^ T cells are known to have the ability to directly eliminate tumor cells, making them the preferred immune cells for targeting tumors (Farhood et al., 2019), However, in order to achieve effective anti-tumor immunity, the complementary CD4^+^ T cells are also necessary (Ahrends et al., 2017). Research has shown that aconitine has multiple effects on cells. It induces apoptosis in tumor cells and activates voltage-dependent Na^+^ channels in the thymus, which leads to the binding of Ca^2+^ to downstream molecules and a decrease in free Ca^2+^ in cells. Additionally, aconitine promotes DP toward SPCD4 and promotes thymic T cell development, maturation, and efflux (Lo et al., 2012). It also increases the percentage of T cells in the spleen and mesenteric lymph nodes, as well as the production of IFN-γ in the spleen and draining lymph nodes, resulting in anti-tumor effects (Zhang, 2021). When aconitine reaches a certain dosage, it can decrease the percentage of Treg obtained by the organism. However, at low doses, not only does it not reduce the percentage of Treg (Cheng et al., 2020), but it can even encourage the differentiation of peripheral blood T cells into Treg (Cheng, 2019). This is done to suppress the function of CD8^+^ T cells, which ultimately leads to a lower thymic index. Immune cells produce cytokines TNF-α, IL-1β, and IFN-γ, which play crucial roles in immunomodulation and intercellular communication within the immune system. Additionally, these cytokines are essential for the differentiation of memory T cells (Castaneda-Delgado et al., 2017). During tumor development, the body exerts its immune function mainly by regulating the secretion of cytokines (Jiang et al., 2022). Aconitine alkaloids have been shown to promote the secretion of cytokines like TNF-α, IL-1β, and IFN-γ in cancer model mice, thereby enhancing the immune function of the body (Wu et al., 2021).

### 4.4 Regulatory genes related to autophagy and apoptosis

Autophagy is a cellular process where cells engulf their own cytoplasmic proteins or organelles, encapsulate them into vesicles, and fuse with lysosomes to form autolysosomes. This process allows for the degradation of the encapsulated contents, which satisfies the metabolic needs of the cell and facilitates the renewal of specific organelles. Autophagy is closely linked to cell growth, apoptosis, and internal environmental homeostasis (Ferro et al., 2020) (Glick et al., 2010) (Maiuri et al., 2007). Autophagy is a self-degrading system that is conserved across species and plays a crucial role in maintaining cellular homeostasis during periods of stress (Onorati et al., 2018). However, when autophagy becomes dysregulated, it can have significant implications for human health and disease. Research suggests that inhibiting autophagy may be a promising therapy for advanced cancers. It is important to note that autophagy is not a singular process, but rather requires interaction with various signaling pathways. Furthermore, it is closely linked to apoptosis (Chude and Amaravadi, 2017; Li et al., 2017) (Maheswari et al., 2018). Bcl-2 is the anti-apoptotic gene that is most strongly linked to apoptosis, while Bax is a pro-apoptotic gene that opposes Bcl-2 (Korsmeyer et al., 1993; Cory and Adams, 2005). During the apoptosis signaling process, the Bcl-2 and Bax genes can regulate the activity of the Caspase-3 gene, which in turn controls the apoptotic process via the mitochondrial pathway (Yaidikar and Thakur, 2015). Caspase-3 plays a crucial role in the apoptotic process as the major terminal shear enzyme and the primary executor. Upon initiation of apoptosis, Caspase-3 protein is activated, and its activating protein, Active Caspase-3 protein, can specifically shear DNA. This prompts cytoplasmic coagulation and nucleic acid activation, ultimately leading to apoptosis (Kuper et al., 2011). In contrast, the results of several papers included in this study, the upregulation of Bax expression and the downregulation of Bcl-2 expression demonstrate that aconitine can promote the expression of apoptotic genes and cause excessive autophagy in cancer cells, which leads to apoptosis. In contrast to other papers examined in this study, the findings indicated that aconitine could promote the expression of apoptotic genes/proteins and excessive autophagy in cancer cells by upregulating Bax expression and downregulating Bcl-2 expression, ultimately leading to apoptosis.

### 4.5 NF-κB signal pathway

Generally, the Bax and Bcl-2 genes work together to maintain a balanced state and facilitate a normal apoptotic process (Siddiqui et al., 2015; Warren et al., 2019). The anti-apoptotic effect of cells is initiated through the activation of a positive cascade of amplified signaling pathways, which are primarily controlled by NF-κB mediated by different survival factors via receptor kinases (Sonenshein, 1997; Dolcet et al., 2005).

Since incorporation of literature demonstrates that aconitine could upregulate Bax and downregulates Bcl-2 expression. Additionally, studies by Ren and Jia demonstrate that aconitine can downregulate the expression of NF-κB pathway (Ren and Zeng, 2008; Jia and Zhang, 2011). Based on this information, it is reasonable to speculate that aconitine achieves its antitumor efficacy through NF-κB signaling pathways. A possible mechanism of aconitine action on the NF-κB signaling pathway were illustrated in Figure 11B.

Under non-activating conditions, NF-κB exists in an inactive form within the cell plasma, and upon extracellular stimulation (e.g., viral or bacterial infection, UV irradiation, etc.), the NF-κB signaling pathway begins to activate (Wu and Kral, 2005). This activation occurs when extracellular signaling factors bind to receptors on the cell membrane, initiating a cascade of downstream responses. The receptor protein receives stimulation and activates I κB kinase (IKK) first (Oeckinghaus and Ghosh, 2009). Intracellular NF-κB-I κB complex is phosphorylated by IKK, which allows for the modification and degradation of I κB, resulting in the release of NF-κB dimers (Hacker and Karin, 2006). These free NF-κB dimers enter the nucleus to bind to genes containing NF-κB binding sites and initiate the transcriptional process (Legrand-Poels et al., 1998). Additionally, NF-κB activates the expression of the I κBα gene, and newly synthesized I κBα re-inhibits NF-κB activity (Baeuerle and Henkel, 1994; Smale, 2012). NF-κB plays a crucial role in regulating cellular responses due to its ability to quickly activate as a master transcription factor without the need for new protein synthesis. It acts as the first responder to harmful stimuli within cells. Several studies (Gilmore et al., 2002; Ali and Mann, 2004; Vasudevan et al., 2004; Clarke et al., 2005) have shown that NF-κB inhibits apoptosis through three primary pathways: ① NF-κB plays a role in both self and other cell apoptosis through the regulation of cytokines; ②NF-κB inhibits apoptosis by inducing or upregulating anti-apoptotic genes; and ③NF-κB inhibits apoptosis by inducing TRAF and IAP.

The NF-κB pathway has a number of known activators, including TNF-α (Liu et al., 2021), interleukin cytokines (He and Karin, 2011), chemokines (Zhao et al., 2021), and colony-stimulating factors (Schreck and Baeuerle, 1990). In addition, some anti-inflammatory molecules such as zinc finger protein (Ye et al., 2019; Zhang et al., 2021), HO-1 (Kim et al., 2019; Saha et al., 2020) and molecules related to apoptosis (TRAF-1, IAP1/IAP2, TRAF1/TRAF2) are also regulated by NF-κB (Zusso et al., 2019; Efferth and Oesch, 2021).

The NF-κB pathway’s anti-apoptotic mechanism involves inhibiting the activation of caspase-8, which in turn inhibits downstream caspase-3. This inhibition is achieved by regulating the expression of TRAF1, TRAF2, c-IAP1, and c-IAP2. Inclusion studies indicate that aconitine is likely to promote the expression of the Bax gene and suppress that of the Bcl-2 gene by regulating the NF-κB signaling pathway. Additionally, aconitine regulates the expression of cell cycle proteins, thereby inhibiting tumor cell proliferation and inducing apoptosis. The anti-apoptotic mechanism of NF-κB is to inhibit the activation of caspase-8 (Dohrman et al., 2005; Schneider et al., 2017) and thus downstream caspase-3 (Luo et al., 2019; Sangaran et al., 2021; Sun et al., 2021) by regulating the expression of TRAF1, TRAF2, c-IAP1, and c-IAP2. Inclusion studies suggested that aconitine was likely to promote the expression of the Bax and suppress that of the Bcl-2 by regulating the NF-κB signaling pathway. Additionally, aconitine could regulate the expression of cell cycle proteins, thereby inhibiting tumor cell proliferation and inducing apoptosis.

## 5 Conclusion

Although aconitine is highly toxic, it has a remarkable anti-tumor effect. Its mechanism of action is complex, but it inhibits tumor cell proliferation and induces apoptosis. Aconitine activates voltage-dependent Na^+^ channels in the thymus, promoting thymic T cell development, while also inhibiting Bcl-2 gene expression and activating the downstream gene Caspase-3 to promote tumor cell apoptosis through the regulation of the NF-κB signaling pathway.

Studies have shown that aconitine is highly toxic, with lethal doses (LD_50_) of 0.2702 ± 0.002 mg/kg in mice, respectively (Zhou et al., 1984). Although aconitine is highly toxic, it has a remarkable anti-tumor effect. It inhibits tumor cell proliferation and induces apoptosis with a complex mechanism, which may inhibit tumor cells by activating voltage-dependent Na^+^ channels in the thymus and promoting thymic T cell development on the one hand. on the other hand, it inhibits Bcl-2 expression and activates the downstream Caspase-3 to promote tumor cell apoptosis by regulating the NF-κB signaling pathway. However, the precise mechanism of aconitine remains unexplored in both preclinical and clinical trials, whether *in vivo* or *in vitro*. At the same time, the challenge of regulating the dosage of aconitine in isolation during clinical trials has led to its frequent use as part of a combination drug in multidrug resistance trials for certain drug-resistant medications. But there are limited *in vitro* and *in vivo* preclinical and clinical studies on this subject, making it a key area of investigation for advancing the clinical application of aconitine.

## Data Availability

The original contributions presented in the study are included in the article/supplementary material; further inquiries can be directed to the corresponding author.
